# Molecular Mechanisms of Zhizhu Kuanzhong Capsule in the Treatment of Co-Morbid Anxiety and Depression of Functional Dyspepsia: Network Pharmacology, Molecular Docking and In Vivo Validation

**DOI:** 10.3390/biomedicines14040867

**Published:** 2026-04-10

**Authors:** Jing He, Ruiyun Wang, Pengcheng Yang, Zhuanglong Xiao, Tao Bai, Xiaohua Hou, Lei Zhang

**Affiliations:** 1 Division of Gastroenterology, Union Hospital, Tongji Medical College, Huazhong University of Science and Technology, Wuhan 430022, China; hejing199909@163.com (J.H.); yangpc@hust.edu.cn (P.Y.); xzlcns1002@126.com (Z.X.); drbaitao@126.com (T.B.); 2 Department of Gerontology, Union Hospital, Tongji Medical College, Huazhong University of Science and Technology, Wuhan 430022, China; wangruiyun525@126.com

**Keywords:** functional dyspepsia, anxiety, depression, monoaminergic system, hypothalamic–pituitary–adrenal axis

## Abstract

**Objective**: ZhiZhu Kuanzhong (ZZKZ) capsule, a Chinese herbal extract, is extensively employed for the clinical management of functional dyspepsia (FD) in China. This study aimed to elucidate the therapeutic efficacy and underlying mechanisms of ZZKZ on the co-morbidity of anxiety and depression of FD. **Methods**: The FD model was established in Sprague–Dawley rats via neonatal gastric irritation with 0.1% iodoacetamide. Subsequently, FD rats were gavaged with ZZKZ or fluoxetine. Depression-like behaviors were evaluated using the sucrose preference test (SPT) and forced swimming test (FST), while anxiety-like behaviors were assessed via light-dark box (LDB) and open field tests (OFTs). Network pharmacology and molecular docking were conducted to explore the mechanisms of ZZKZ’s action. Hippocampal levels of monoamine neurotransmitters and monoaminergic system components were evaluated by HPLC and RT-qPCR, respectively. Serum concentrations of HPA axis hormones were determined by ELISA. **Results**: ZZKZ administration reversed the deficits in body weight gain and food intake in FD rats. Behaviorally, ZZKZ increased sucrose consumption in SPT and prolonged swimming duration in FST, and it increased duration and entries into the central zone in OFT. According to the prediction of network pharmacology, ZZKZ treatment elevated hippocampal levels of 5-HT/NE/DA, increased expression of TPH2/TH, and decreased expression of MAO_A_/SERT in FD rats. Molecular docking further confirmed high-affinity binding between core ingredients of ZZKZ and TPH2/TH/MAO_A_/SERT. Moreover, ZZKZ administration attenuated the stress-induced elevation of serum CRH/ACTH/CORT. **Conclusions**: ZZKZ effectively ameliorates the disordered gut–brain interaction and mitigates anxiety-like and depression-like behaviors, which might be modulated by the hippocampal monoaminergic system and hypothalamic–pituitary–adrenal axis response.

## 1. Introduction

Functional dyspepsia (FD) represents one of the most prevalent functional gastrointestinal disorders (FGIDs) globally [1], with an estimated worldwide prevalence of 16% and figures reaching as high as 18–45% in China [2]. Clinically, FD is characterized by a constellation of symptoms including postprandial fullness, early satiety, epigastric pain, and/or epigastric burning [3], all of which are closely associated with disordered gut–brain interactions as defined by the Rome IV criteria [4]. Patients with FD commonly exhibit psychological comorbidities such as anxiety, depression, and sleep disturbances [5,6], highlighting the nature of FD as both a physical and a mental disorder. Notably, the prevalence of anxiety and depression symptoms is significantly elevated in patients with refractory FD, with rates reaching 61.5% and 63.3%, respectively [7]. Furthermore, a substantial body of evidence indicates that psychological stress and emotional abnormality can profoundly disrupt the brain–gut interaction, leading to gastrointestinal inflammation, dysmotility, and visceral hypersensitivity, all of which considerably contribute to the development of FD [8,9]. Consequently, the gut–brain interaction is recognized as playing a pivotal role in the pathophysiology of FD.

Investigations into the neurobiology of anxiety and depression have consistently implicated several key pathways, prominently featuring the monoaminergic system and the hypothalamic–pituitary–adrenal (HPA) axis [10,11,12], and pharmacotherapies targeting these pathways are established in clinical practice [13,14]. In the context of FD, functional neuroimaging studies have revealed aberrant activity within limbic system regions—areas critical for visceral sensation, emotional processing, and cognition [15]. Notably, the hippocampus, a vital component of the limbic system, is essential for memory, mood, and cognitive function [16]. Studies have indicated that structural and functional alterations in the hippocampus, such as volume reduction [17] and inadequate levels of monoamine neurotransmitters serotonin (5-HT)/norepinephrine (NE)/dopamine (DA) [18], are closely linked to the onset and progression of depression and anxiety. Additionally, hyper-responsiveness of the HPA axis to stress is considered a significant factor in the psychological manifestations of FD [19]. Within the framework of the gut–brain interaction theory, monoamines (particularly 5-HT) not only regulate mood but also modulate gastrointestinal motility and visceral sensation [20,21,22]. Similarly, stress-induced hyperactivation of the HPA axis and subsequent corticotropin-releasing factor (CRF) release directly contribute to delayed gastric emptying, gastric hyperalgesia, and gastrointestinal mucosal inflammation [23,24,25], which are core pathological features of FD. Therefore, the restoration of perturbed monoaminergic system signaling and HPA axis homeostasis emerges as a mechanistically sound and potentially transformative approach for the treatment of co-morbid anxiety and depression of FD.

Currently, specific pharmaceutical treatment for FD remains elusive, particularly for the large subset of FD patients with co-morbid anxiety and depression. Emerging research has highlighted the multi-target effects of Chinese herbal medicines in effectively treating both the gastrointestinal and psychological facets of FGIDs [2,26,27]. One such herbal remedy is the Zhizhu Kuanzhong (ZZKZ) formula, a traditional Chinese medicine that has been widely used for the treatment of FGIDs in China [28,29]. The ZZKZ capsule is an approved modern adaptation of classic ZZKZ formulations: the Zhizhu decoction in the “Synopsis of the Golden Chamber’’ (Han Dynasty, 3rd century) and the Zhizhu pill in the “Differentiation on Endogenous” (Jin Dynasty, 1115–1234) [30]. ZZKZ capsule is composed of four key herbs: immature fruit of *Citrus aurantium* L., rhizome of *Atractylodes macrocephala* Koidz., roots of *Bupleurum chinense* DC., and mature fruit of *Crataegus pinnatifida* Bunge [30]. Our previous research has indicated that ZZKZ improves gastric motility and attenuates visceral hypersensitivity through the gastric 5-HT system to treat FD at the peripheral level [31], while also conferring benefits to mental state, as others reported [32]. However, a critical knowledge gap remains: the therapeutic efficacy and potential mechanisms of ZZKZ on the central co-morbidity associated with FD, framed within the gut–brain interaction theory, have not been fully elucidated.

In the present study, an iodoacetamide-induced FD rat model was established, and the anxiety- and depression-like behaviors of FD rats were evaluated following ZZKZ administration. Our investigation specifically focused on unraveling the mechanisms of the anti-anxiety/anti-depression effects of ZZKZ by integrating network pharmacology and molecular docking with empirical examinations of the hippocampal monoaminergic system and the HPA axis response to stress.

## 2. Materials and Methods

### 2.1. Herbal Drugs and Fingerprint

ZZKZ capsule contains four herbal ingredients (Table 1): the immature fruit of *Citrus × Aurantium* L. (Zhi Shi), rhizome of *Atractylodes Macrocephala* Koidz. (Bai Zhu), root of *Bupleurum Chinense* DC. (Chai Hu) and mature fruit of *Crataegus Pinnatifida* Bunge (Shan Zha). All products were provided by ShuangRen Pharmaceuticals Co. Ltd. (Shanxi, China), Lonch Group, China (drug approval number: Z20020003; batch numbers: 141042, 150106, 150422, 150838, 150840, 151255, 160401, 160402, 160403, and 160504), and authenticated according to the Chinese Pharmacopeia (2020 version). The duration of this study is from 12 April 2017 to 11 May 2025.

**Figure 1:**
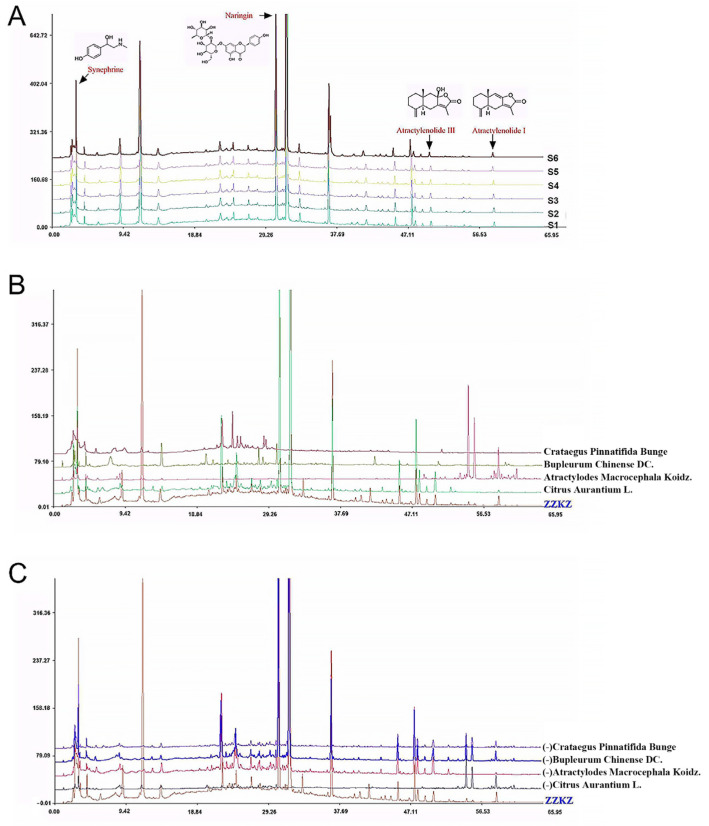
HLPC fingerprint of ZZKZ. (**A**) Chromatogram of different batches of ZZKZ capsules. Twenty characteristic peaks were detected in ZZKZ, which were attributed to the four various herbs, including synephrine (1), naringin (7), atractylenolide III (19), and atractylenolide I (20). (**B**) HLPC spectrum for every single ingredient of ZZKZ. (**C**) HLPC spectrum for the ZZKZ compound lacks every single ingredient.

This research was conducted according to the ConPhyMP guidelines [33] to ensure reproducibility and accurate interpretations of studies using ZZKZ aqueous extract (drug extract ratio: 18.5:1). The fingerprint of ZZKZ was plotted via high-performance liquid chromatography (HPLC) analysis, as shown in Figure 1. In brief, 1 g of ZZKZ was dissolved in 25 mL of aqueous methanol (80%, *v*/*v*) and boiled in a water bath for 1.5 h, and was cooled naturally. The extracted solution was filtered twice through a 0.45 μm microporous membrane, and the continued filtrate was used as the test solution. HPLC analysis was performed on an Agilent 1260 Infinity HPLC system (Agilent technologies, Santa Clara, CA, USA), with a YMC-C18 column (250.0 × 4.6 mm, 5 μm) for separation. The column temperature was maintained at 30 °C, the flow rate was 1.0 mL/min, the detection wavelength was set to 276 nm, and the injected sample volume was 10 µL. The mobile phase consisted of acetonitrile (A) and purified water (B), using the following gradient elution: 0–5 min, 2% A; 5–10 min, 5% A; 10–35 min, 7% A; 35–50 min, 35% A; 50–65 min, 65% A; and >65 min, 100% A. HPLC chromatograms of various batches of ZZKZ capsules (Figure 1A) and the spectrum for each single ingredient of ZZKZ (Figure 1B) as well as the ZZKZ compound (Figure 1C) were analyzed and plotted. Twenty characteristic peaks were identified in the ZZKZ capsule, attributed to four various herbs: synephrine (1), naringin (7), atractylenolide III (19), and atractylenolide I (20).

### 2.2. Animals

Male Sprague–Dawley rats (aged 10 days, weighing 14–18 g; Experimental Animal Center, Tongji Medical College, Huazhong University of Science and Technology, Wuhan, China) were used. The neonatal rats and the mother rats were housed in specific-pathogen-free conditions with unlimited access to food and drinking water, with a temperature of 23 °C, a 12/12 h light-dark cycle, and a humidity of 50 ± 7%. The pups were weaned at the age of 3 weeks and were housed 3 pups per cage. Each rat was considered an experimental unit. The investigators who performed the outcome assessment were blinded to group allocation. All animal experiments complied with the Guide for the Care and Use of Laboratory Animals and were approved by the Institutional Animal Care and Use Committee, Tongji Medical College, Huazhong University of Science and Technology ([2016] IACUC Number: S758).

### 2.3. FD Models and Treatments

**Figure 2:**
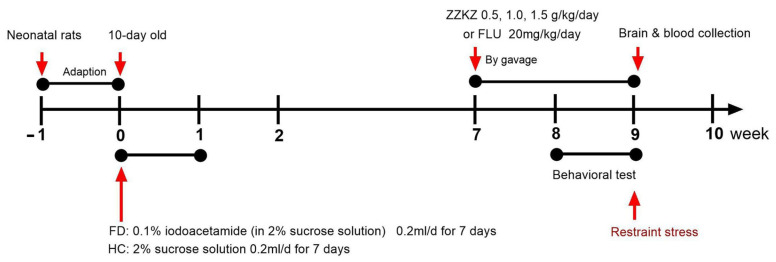
**Time schedule of the experimental design.**

The time schedule of the whole experimental design is shown in Figure 2. FD was induced by neonatal gastric irritation in male Sprague–Dawley rats [34,35]. Ten-day-old rats were treated with 0.1% iodoacetamide (soluble in 2% sucrose solution) (Sigma-Aldrich, St. Louis, MO, USA, I6125) or vehicle at a fixed administration volume of 0.2 mL/d by gavage for 7 consecutive days. The FD-like conditions were developed in the adult rats (8 weeks old), including gastric sensorimotor dysfunction and depression/anxiety-like behavior. At the 7th week, the FD rats were treated with ZZKZ at a low (ZZKZ-L, 0.5 g/kg), medium (ZZKZ-M, 1.0 g/kg), or high dose (ZZKZ-H, 1.5 g/kg) per day by gavage for 2 weeks, while rats treated with fluoxetine (FLU, 20 mg/kg/day) (MedChemExpress, Monmouth Junction, NJ, USA, HY-B0102) were conducted as treatment controls. Subsequently, the rats were subjected to behavioral assessments, followed by sample collection for further detection. A total of 46 male Sprague–Dawley rats were randomly assigned to the healthy control (HC) group *(n* = 6) and various experimental groups (*n* = 8 per group) using a computer-generated random number sequence. The smaller size of the HC group was specifically intended to adhere to the 3R principles (reduction) by minimizing the sacrifice of healthy animals, given the lower biological variance typically observed in healthy cohorts.

To assess the HPA response to a minor stressor, rats were subjected to an acute restraint stress [36]: the rats were immobilized in a cylindrical plastic tube with adequate ventilation, restricting movement to the head and upper limbs only. Following a 60-min stress period. Then, all rats were anesthetized using a 50 mg/kg dose of sodium pentobarbital administered intraperitoneally, and the blood samples were collected for hormonal analysis. Thereafter, rats were quickly euthanized by rapid cervical dislocation. After 10 s of rapid cervical dislocation, rats were judged to be dead if they stopped breathing and had no response to limb and head stimulation.

The animal procedures were conducted by Lei Zhang, Ruiyun Wang, and Jing He from 12 April 2017 to 19 May 2021 and from 1 September 2022 to 11 May 2025 in the Laboratory Animal Center of Huazhong University of Science and Technology. The experimental protocols, care, and handling of animals used in this study were approved by the Institutional Animal Care and Use Committee, Tongji Medical College, Huazhong University of Science and Technology, in accordance with the guidelines of the International Association for the Study of Pain (IASP).

### 2.4. Behavioral Assessments

The sucrose preference test (SPT) and forced-swimming test (FST) were utilized to assess depression-like behaviors, while the open field test (OFT) and light-dark box test (LDB) were conducted to evaluate anxiety-like behaviors [35,37]. Rats were acclimated to the test room for at least 1 h prior to each test. Behavioral tests were conducted in sequential order of SPT, OFT, LDB, and FST with a one-day interval between each test. The researchers responsible for behavioral tests were different from those who conducted data analysis.

#### 2.4.1. Sucrose Preference Test (SPT)

The rats were singly housed in a cage with two bottles containing 1% sucrose solution and tap water, respectively, and trained to drink sucrose water for 48 h. The position of the two bottles was exchanged every 12 h to prevent possible effects of side preference in drinking behavior. After the training session, rats were deprived of food and drinking water for 12 h. Then, the rats had access to two bottles containing the same amount of 1% sucrose water or pure water, and the water consumption was recorded after 12 h. The sucrose preference was calculated by the formula: sucrose preference (%) = sucrose intake/(sucrose intake + water intake) × 100%.

#### 2.4.2. Open Field Test (OFT)

The open field apparatus consisted of an uncovered square box (100 cm × l00 cm × 60 cm) with black walls and a white bottom. The arena was composed of 100 equal squares. The device is placed in a quiet experimental room and in a shading curtain with uniform brightness. The rat was gently placed in the center of the field and was allowed to explore in the arena for 5 min. Movement trajectory was recorded by a video camera mounted above the open field and analyzed via the video-tracking system. The horizontal movement score (the number of rats crossing the bottom grid, 1 point per grid), the vertical movement score (the number of rats standing upright, 1 point for each time), and the percentage of time spent in the center were recorded. Locomotor activity and total time spent in the center were analyzed in EthoVision XT 8.0 (Noldus, Wageningen, The Netherlands).

#### 2.4.3. Light-Dark Box Test (LDB)

The light-dark box consisted of two equally sized compartments (30 cm × 30 cm × 40 cm) connected by an 8 cm × 8 cm door. The dark box was painted black and covered with a black lid, and the light box was opaque and remained uncovered during the test. The tests were performed between 9 am and 3 pm, and rats were adapted in the test room for 30 min prior to testing. Then, the rat was placed in the center of the light box with its back to the dark box and allowed to explore freely in the apparatus for 10 min. The movements of the animals were tracked by a video camera positioned above the center of the light-dark box and analyzed using the video tracking system (Noldus Information Technology, Wageningen, The Netherlands). The data of behavior analysis included the percentage of time spent in the light box and dark box, and the number of transitions, which are defined as at least half of the animal’s body moving from one chamber to the next.

#### 2.4.4. Forced Swimming Test (FST)

The rats were adapted in a clear plastic water tank (50 cm × 50 cm × 60 cm) filled with 30 cm of 25 ± 1 °C water for a 15 min swim prior to testing. After 24 h, each rat was forced to swim in the water tank for 5 min. Both test sessions were recorded by a video camera and analyzed using Ethovision XT 8.0 software (Noldus, Wageningen, The Netherlands) and the videotapes were scored by a blind examiner using a time-sampling technique: the behavior at the end of each 5 s period was categorized as one of the following: (1) immobility: the rat remained floating in the water without struggling and made only those movements necessary to keep its head above water; (2) swimming: the rat displayed active swimming motions, more than necessary to merely maintain its head above water, e.g., moving around in the cylinder; (3) climbing: the rat displayed active movements with its forepaws in and out of the water, usually directed against the walls.

### 2.5. High-Performance Liquid Chromatography (HPLC)

HPLC analysis was applied to detect the concentrations of monoamine neurotransmitters, including 5-hydroxytryptamine (5-HT), dopamine (DA), norepinephrine (NE), and the metabolic products 5-hydroxyindoleacetic acid (5-HIAA), 3,4-Dihydroxyphenylacetic acid (DOPAC), and homovanillic acid (HVA) in the hippocampus. In brief, hippocampus tissues were homogenized using an ultrasonic cell disruptor. Then, 30 μL of hippocampus supernatant or standard was mixed and vortexed with 15 μL of benzoyl chloride (2% in acetonitrile, *v*/*v*) (Sigma-Aldrich, B12695) and 15 μL of borate buffer (sodium tetraborate, 100 mM) (Sigma-Aldrich, 89293). Then, HPLC measurement was performed on an Agilent 1260 Infinity HPLC-Chip/MS System (Agilent technologies). The flow rate was 600 μL/min, the sample injection volume was 20 µL, and the column temperature was maintained at 30 °C. Data were acquired, calibrated, and quantified with Agilent ChemStation (B.03.01), and the concentration (neurotransmitter/tissue, ng/mg) of each sample was calculated.

### 2.6. Real-Time Quantitative PCR Analysis (RT-qPCR)

RT-qPCR analysis was used for evaluating the gene expression of key molecules in the synthesis (tryptophan hydroxylase 2, TPH2; tyrosine hydroxylase, TH), transport (serotonin transporter, SERT; dopamine transporter, DAT; noradrenaline transporter, NET), and degradation (monoamine oxidase A, MAO_A_; monoamine oxidase B, MAO_B_) of monoamine neurotransmitters. In brief, total RNA of hippocampal tissues was extracted using a Trizol Reagent (Invitrogen, Carlsbad, CA, USA, 15596018CN). A two-step real-time quantitative PCR was subsequently performed. A PrimeScript RT Master Mix Kit (TaKaRa, Shiga, Japan, RR036A) was used to synthesize cDNA, and a QuantiTest SYBR Green PCR Kit (QIAGEN, Venlo, The Netherlands, Cat no. 204145) was used for RT-qPCR on a ROCHE LightCycler^®^ 480 System (ROCHE, Basel, Switzerland) according to the manufacturer’s instructions. The primer sequences are shown in Table 2, in which endogenous GAPDH was used as a normalization reference. The relative expression of the mRNA species was quantified using the 2^−∆∆CT^ method.

### 2.7. Enzyme-Linked Immunosorbent Assay (ELISA)

Serum levels of HPA-axis-related hormones at baseline and post-stress conditions were detected by the ELISA method. Specifically, the concentrations of serum corticotropin-releasing hormone (CRH; BioVendor, Brno, Czech Republic, catalog no. RSCYK131R), adrenocorticotropic hormone (ACTH; Abnova, Taiwan, China, catalog no. KA0917), and corticosterone (CORT; Arigo Biolab, Taiwan, China, catalog no. ARG80652) were quantified with specific ELISA kits according to the manufacturer’s instructions.

### 2.8. Network Pharmacology Analysis

The core ingredients (synephrine, naringin, atractylenolide III, and atractylenolide I) of ZZKZ identified by HPLC were imported into the SwissTargetPrediction database (http://swisstargetprediction.ch/) (assessed on 10 April 2025) [38] to obtain ZZKZ’s corresponding targets (probability > 0). Next, the GeneCards database (https://www.genecards.org/) (assessed on 10 April 2025) [39] was searched for anxiety/depression-related targets. Because the primary objective of our network pharmacology analysis was to comprehensively identify potential therapeutic pathways rather than prematurely isolating specific molecular targets, no strict Relevance Score filters were applied at this initial retrieval stage. This strategy prevented the premature exclusion of molecules critical to pathway enrichment. Subsequently, the intersected targets of the above three lists were then analyzed using the STRING database (https://string-db.org/) (assessed on 15 April 2025) [40] and TRRUST database (https://www.grnpedia.org/trrust/Network_search_form.php) [41] (assessed on 16 April 2025) to construct a protein–protein interaction (PPI) network and an ingredients-protein-transcription factor network. The resulting PPI network was subsequently visualized with the help of Cytoscape 3.10.2 software [42], and the MCODE plugin was then employed to analyze and evaluate the degree values for each target. Targets that exhibited degree scores above three and equal to three were selected and considered as hub genes of ZZKZ’s effects. The key transcription factor was identified by the number of interactions between the transcription factor and overlapping genes. Subsequently, the targets related to ZZKZ and anxiety/depression were subjected to Gene Ontology (GO) enrichment analysis and Kyoto Encyclopedia of Genes and Genomes (KEGG) enrichment analysis using the ‘clusterProfiler’ package and ‘ggplot2’ package in R (version 4.4.3). To control the type-I error rate during large-scale genomic testing, the Benjamini–Hochberg (BH) method was employed for multiple testing correction, and an adjusted *p* value < 0.05 was defined as statistical significance. All network pharmacology analyses were conducted in the “*homo sapiens*” species by Jing He from 1 September 2022 to 11 May 2025.

### 2.9. Molecular Docking

Molecular docking was used to validate abnormal monoaminergic molecules (TPH2/TH/MAO_A_/SERT) identified by the above rat experiments. First, we searched the core ingredient compounds in the PubChem database (https://pubchem.ncbi.nlm.nih.gov/) (assessed on 1 May 2025) and the monoaminergic molecules in the Protein Data Bank database (https://www.rcsb.org/) (assessed on 1 May 2025) [43], and the 3D structures were exported. Then, the ligands of the monoaminergic molecules were removed using Pymol software (Version 2.6), and the progress of dehydration, hydrogenation, charge editing, root addition, and docking progress were monitored using AutoDock Vina (Version 1.1.2) [44]. The grid box is designed to fully encompass the receptor protein, and ten independent docking runs were executed for each protein-ligand pair. The binding potential was assessed by an affinity score, and the optimal docking model (lowest binding energy) was then selected to import into Pymolsoftware for analysis and visualization. The molecular docking analysis was conducted by Jing He from 1 September 2022 to 11 May 2025.

### 2.10. Data Expression and Statistical Analysis

All data were shown as mean ± standard deviation (SD), except for quantitative data of hippocampal monoamine neurotransmitters and monoaminergic system components in HPLC/RT-qPCR experiments, which were presented as median and extreme values in a box plot. Prior to comparative analysis, data distribution normality and homogeneity of variance were rigorously assessed using the Shapiro–Wilk test and Levene’s test, respectively. Since all continuous data met the assumptions of normal distribution and equal variance, statistical significance among multiple groups was determined exclusively using a one-way analysis of variance (ANOVA) followed by the Least Significant Difference (LSD) post hoc test. A *p* value less than 0.05 was considered statistically significant. SPSS 21.0 software was used for statistical analysis, and GraphPad Prism5 software was used for drawing.

## 3. Results

### 3.1. ZZKZ Administration Increased the Body Weight and Food Intake of FD Rats

Consistent with the features of the FD model, untreated FD rats exhibited significantly impaired growth, characterized by lower body weight and a reduced rate of weight gain compared to the healthy control (HC) group, and administration of ZZKZ or fluoxetine effectively reversed this growth deficit (Table 3). Specifically, the rates of body weight increase in the 2-week treatment period were significantly elevated in the FD + FLU group (14.67 ± 4.52%) and FD + ZZKZ groups (L 17.13 ± 7.92%, M 18.94 ± 5.85%, and H 15.44 ± 8.46%), compared to the untreated FD group (6.54 ± 2.77%, all *p* < 0.05). Additionally, untreated FD rats displayed reduced 24 h food intake, and ZZKZ administration significantly promoted the food consumption, particularly at the medium and high doses (Table 3).

### 3.2. ZZKZ Improved the Depression-like Behavior in FD Rats

**Figure 3:**
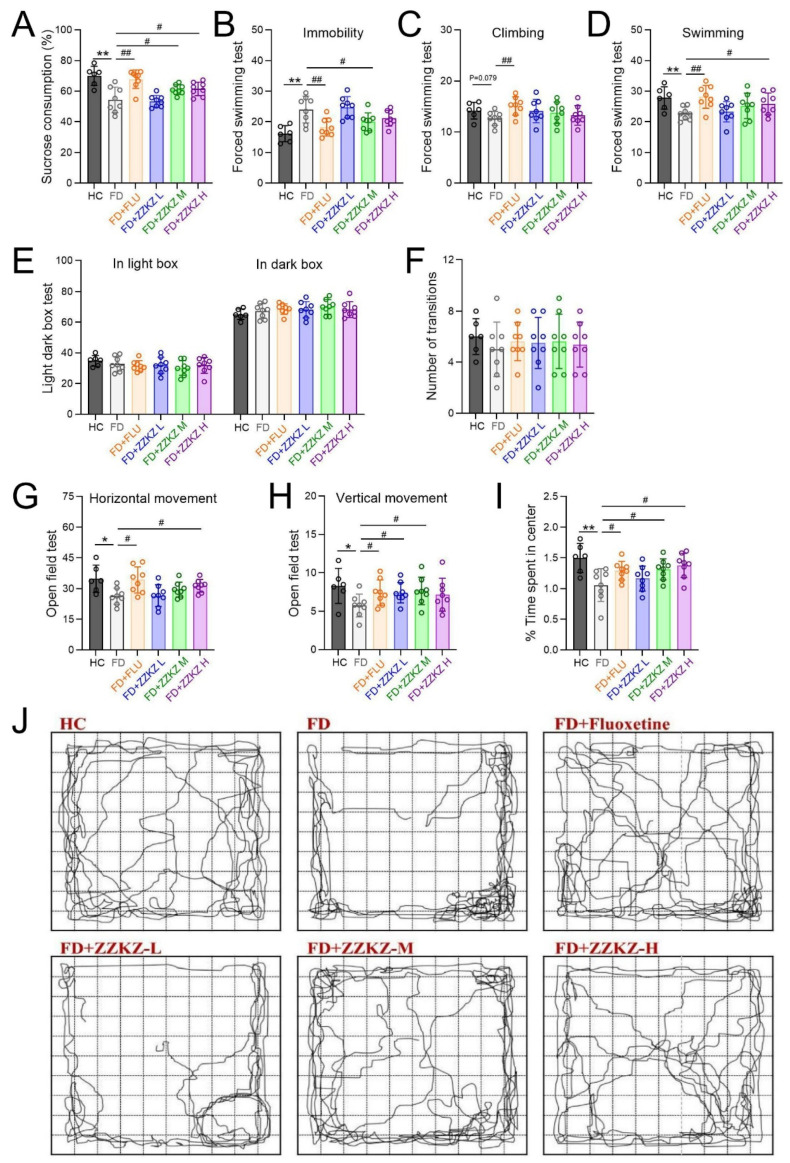
**ZZKZ administration alleviated the depression-like and anxiety-like behavior in FD rats.** The depressive-like behavior of FD rats was evaluated via SPT and FSTs. (**A**) The sucrose consumption rate in the sucrose preference test. (**B**–**D**) The counts of immobility, swimming, and climbing movements in the forced swim test. The anxiety-like behavior of FD rats was evaluated via LBD and OFTs. (**E**) The percent of time spent in the light and the dark box, and (**F**) the counts of transitions between the two compartments of the light-dark box in the LBD test. (**G**,**H**) The counts of horizontal and vertical movements, and (**I**) the percent of time spent in the center of the open field in OFTs. (**J**) The typical diagrams of movement trajectory in the open field. Data were shown as mean ± SD, *n* = 6–8. * *p* < 0.05 and ** *p* < 0.01 FD group vs. HC group; ^#^ *p* < 0.05 and ^##^ *p* < 0.01 FD + FLU/FD + ZZKZ group vs. FD group.

To assess depression-like behaviors of FD rats, we evaluated anhedonia (loss of pleasure) and behavioral despair (loss of hope) using the SPT and FST, respectively. In the SPT, FD rats showed a reduced sucrose consumption compared to the healthy controls (Figure 3A; 49.91 ± 4.65% vs. 74.98 ± 1.71%, *p* < 0.01), and this deficit was completely reversed by fluoxetine treatment (Figure 3A; 75.74 ± 4.95% vs. 49.91 ± 4.65%, *p* < 0.01) or medium/high dosage of ZZKZ (Figure 3A; M 61.70 ± 1.49% and H 61.63 ± 1.05% vs. 49.91 ± 4.65%, *p* < 0.05). In the FST, FD rats showed increased immobility behaviors (Figure 3B; 26.63 ± 1.16 vs. 16.01 ± 0.67, *p* < 0.01), potentially declined climbing behaviors (Figure 3C; 12.60 ± 0.97 vs. 14.16 ± 0.59, *p* = 0.079), and decreased swimming behaviors (Figure 3D; 20.61 ± 1.59 vs. 27.89 ± 1.16, *p* < 0.01), all of which were alleviated by treating with fluoxetine as well as ZZKZ treatment to some extent.

### 3.3. ZZKZ Improved the Anxiety-Like Behavior in FD Rats

Anxiety-like behaviors of FD rats were assessed using the LDB and OFT. In the LDB, no significant differences were observed between FD and healthy controls in either the time spent in the box or the number of transitions (Figure 3E,F). In the OFT, the FD rats exhibited anxiety-like behavior with significantly decreased horizontal movements (Figure 3G; 25.50 ± 6.38 vs. 39.38 ± 4.53, *p* < 0.05) and vertical movements (Figure 3H; 5.64 ± 2.27 vs. 8.55 ± 2.44, *p* < 0.05), as well as declined time spent in the central zone (Figure 3I; 0.90 ± 0.36 s vs. 1.49 ± 0.33 s, *p* < 0.01) compared to the healthy control. Treatment with medium and high doses of ZZKZ significantly increased the time spent in the central zone compared to untreated FD rats (Figure 3I; M 1.23 ± 0.36 s and H 1.34 ± 0.24 s vs. 0.90 ± 0.36 s, *p* < 0.05), which indicated a compromised anxiety-like behavior. Moreover, more active and complex movement trajectories with increased entries into the central zone were observed in ZZKZ-treated FD rats (Figure 3J). The synchronized improvements in anhedonia (SPT), behavioral despair (FST), and exploratory inclination (OFT) provide mutually reinforcing evidence, suggesting that ZZKZ exerts a broad-spectrum therapeutic effect on FD-related neuropsychiatric deficits.

### 3.4. Monoaminergic System and Hypothalamic–Pituitary–Adrenal Axis Response Were Identified Through Network Pharmacology Analysis

**Figure 4:**
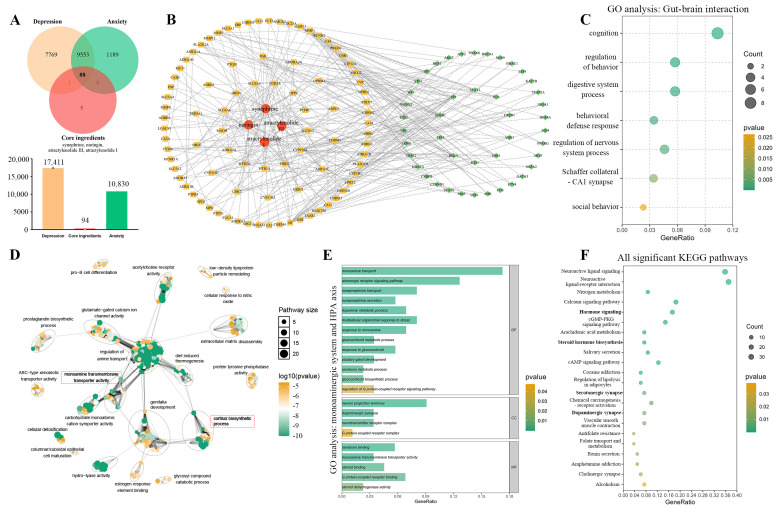
**Network pharmacology analysis of core ingredients of ZZKZ.** (**A**) Venn diagram of the four core ingredients of ZZKZ (synephrine, naringin, atractylenolide III, and atractylenolide I) and anxiety/depression-associated targets. (**B**) Ingredients-protein-transcription factor network. Red circles were core ingredients, yellow squares were common target genes, and the three rings were arranged according to degree score (inner ring > 3, middle ring = 3, outer ring < 3). The green diamond was an associated transcription factor, and the two rings were arranged according to the number of overlapped genes (the inner ring was the top 10 hub transcription factors). The lines represented interactions between core ingredients, target genes, and transcription factors. (**C**) gut–brain interaction associated terms based on GO enrichment analysis of common target genes (all *p* < 0.05). (**D**) visualized network of GO enrichment analysis among all terms with *p* < 0.01. (**E**) associated terms of the monoaminergic system and hypothalamic–pituitary–adrenal axis in GO enrichment analysis (all *p* < 0.05). (**F**) all significant pathways in the KEGG enrichment analysis of common target genes. The bold pathways were related to the monoaminergic system and hypothalamic–pituitary–adrenal axis.

Considering the multi-target effects of traditional Chinese medicine compounds, we subsequently conducted network pharmacology analysis to shed light on the underlying mechanism of ZZKZ’s anti-anxiety/anti-depression effects. Initially, 94 genes relevant to the four core ingredients of ZZKZ (synephrine, naringin, atractylenolide III, and atractylenolide I) were identified utilizing the SwissTargetPrediction database, while 17,411 genes associated with depression and 10,830 genes associated with anxiety were sourced from the GeneCards database (Figure 4A). Subsequently, 88 shared targets were employed to construct an ingredients-protein-transcription factor network with the help of the TRRUST database, combining PPI analysis through the STRING database. The primary targets of the inner ring (degree > 3) include SLC26A3, SLC26A4, CYP19A1, HTR1A, DPP4, DRD2, DRD4, HTR2A, HTR3A, ADRA2A, SLC6A2, PTPRC, MAOB, and the top 10 intermediary transcription factors of ZZKZ, including SP1, RELA, NFKB1, JUN, ESR1, STAT3, ETS1, CREB1, TP53, and YBX1 (Figure 4B). We observed that hub genes in the inner ring (degree > 3) and middle ring (degree = 3) primarily belonged to the monoaminergic system [45,46,47], and others were partly classified into the hypothalamic–pituitary–adrenal axis [48,49]. GO enrichment analysis confirmed the effects of ZZKZ on gut–brain interaction (Figure 4C), and the GO network also indicated the dominant roles of “monoamine transmembrane transporter activity” and “cortisol biosynthetic process” in all significant GO enrichment terms (red dashed box, Figure 4D). Moreover, associated processes and pathways of the monoaminergic system and hypothalamic–pituitary–adrenal axis were significantly enriched in GO enrichment analysis (Figure 4E) and KEGG enrichment analysis (Figure 4F). Therefore, we assumed that the monoaminergic system and hypothalamic–pituitary–adrenal axis were the potential mechanisms of action of ZZKZ on the co-morbidity anxiety and depression of FD.

### 3.5. ZZKZ Administration Modulated Hippocampal Monoaminergic System with Increased Monoamine Neurotransmitters and Enhanced Monoaminergic Effects

**Figure 5:**
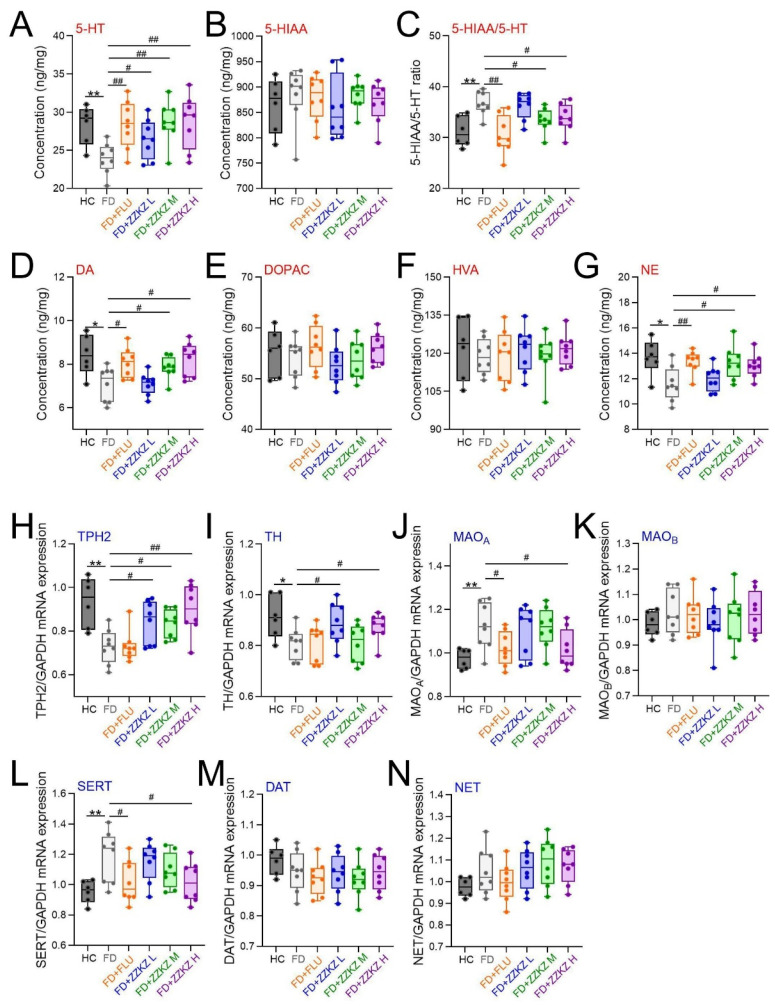
**ZZKZ administration modulated the hippocampal monoaminergic system in FD rats.** The hippocampal levels of monoamine neurotransmitters and metabolic products, including (**A**) 5-Hydroxytryptamine (5-HT), (**B**) 5-hydroxyindoleacetic acid (5-HIAA), (**C**) 5-HIAA/5-HT, (**D**) dopamine (DA), (**E**) 3,4-dihydroxyphenylacetic acid (DOPAC), (**F**) homovanillic acid (HVA), and (**G**) norepinephrine (NE). (**H**,**I**) The expressions of monoaminergic synthesis enzymes, including tryptophan hydroxylase 2 (TPH2) and tyrosine hydroxylase (TH). (**L**–**N**) The expressions of monoaminergic transporters for reuptake, including serotonin transporter (SERT), dopamine transporter (DAT), and noradrenaline transporter (NET). (**J**,**K**) The expressions of monoaminergic degradation enzymes, including monoamine oxidase A (MAO_A_) and monoamine oxidase B (MAO_B_). Data were shown as mean ± SD, *n* = 6–8. * *p* < 0.05 and ** *p* < 0.01 FD group vs. HC group; ^#^ *p* < 0.05 and ^##^ *p* < 0.01 FD + FLU/FD + ZZKZ group vs. FD group.

Next, the function of the monoaminergic system in the hippocampus of FD rats was assessed. The levels of monoamine neurotransmitters, including 5-HT, DA, and NE [50], were significantly lower in FD rats compared with healthy controls (*p* < 0.05), which were significantly elevated by treating with ZZKZ or fluoxetine (Figure 5A,D,G). No significant changes were observed in the levels of the monoamine metabolites such as 5-HIAA, DOPAC, and HVA between the FD group and the FD + ZZKZ group (Figure 5B,E,F). However, the ratio of 5-HIAA/5-HT was increased in the FD group compared to the healthy control, which was significantly decreased by ZZKZ treatment (Figure 5C).

Then, the effects of ZZKZ administration on the expressions of monoaminergic synthesis enzymes (TPH2 and TH; Figure 5H,I), degradation enzymes (MAO_A_ and MAO_B_; Figure 5J,K), and transporters for reuptake (SERT, DAT, and NET; Figure 5L–N) were further detected. The results demonstrated that ZZKZ administration raised the gene expression of TPH2 and TH and decreased the gene expression of MAO_A_ and SERT, which indicated enhanced monoaminergic effects in the hippocampus.

### 3.6. Molecular Autodocking Confirmed the Interaction Between ZZKZ and Monoaminergic System Components

**Figure 6:**
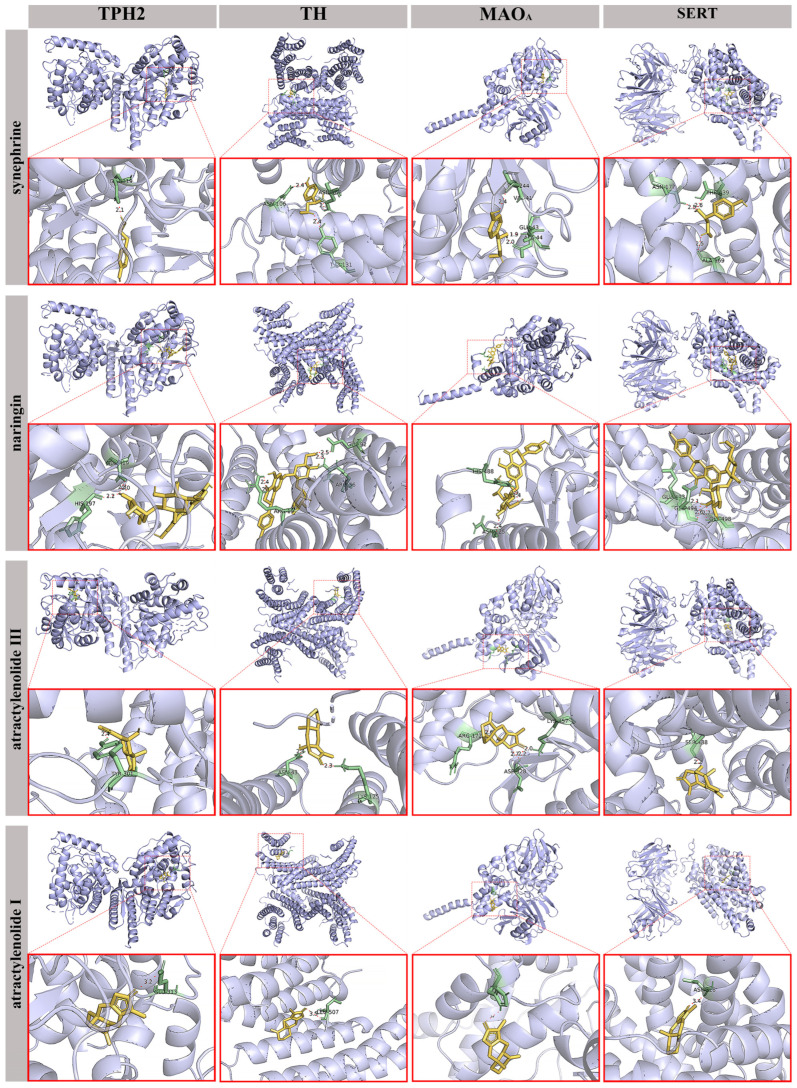
**Molecular autodocking analysis between core ingredients of ZZKZ and monoaminergic system components.** In each model, the light blue structures represented the monoaminergic molecules, while the yellow structures represented the core ingredients. The green zone in monoaminergic molecules represented the binding sites of residues. The red dashed lines represented hydrogen bonds, and the labels in each model showed the residues and the distance of the hydrogen bonds.

Considering the dominant role of the monoaminergic system in ZZKZ effects identified by network pharmacology analysis, we employed molecular autodocking in *homo sapiens* using the core ingredients of ZZKZ and abnormal monoaminergic molecules (TPH2/TH/MAO_A_/SERT) identified by the above rat experiments. It is widely accepted that a smaller Vina score (binding energy) calculated by AutoDock Vina indicates a more stable structure between ligand and receptor, suggesting that the chemical compound is more likely to interact with the protein. Table 4 demonstrated that all core ingredients of ZZKZ could spontaneously bind to selected monoaminergic molecules, and all of them had binding energies less than −5 kcal/mol. Specifically, as visually annotated in Figure 6, molecular docking results also explicitly demonstrated that all core ingredients of ZZKZ established stable intra-molecular interactions with the selected monoaminergic molecules through robust hydrogen-bonding networks. For instance, synephrine formed distinct hydrogen bonds with key residues such as ALA-169/ASN-177/THR-439 of SERT. Similarly, naringin occupied the active pockets by interacting with ARG-19/ARG-56/GLU-92 of TH. The structural analog atractylenolide III also exhibited specific spatial binding with residues like ARG-172/ASP-328/LYS-357 of MAO_A_. These detailed atomic interactions provide compelling structural evidence for their high binding affinities. Based on the docking experiments, we effectively predicted the high affinity and stable binding of ZZKZ to monoaminergic system components and the potential effects of ZZKZ on gut–brain interaction at the human level.

The core ingredients were identified by high-performance liquid chromatography (HPLC) analysis, and the monoaminergic molecules were selected according to the above rat experimental results.

### 3.7. ZZKZ Administration Relieved the HPA Response to Stress with Declined Serum Levels of CRH, ACTH, and CORT

The serum levels of HPA hormones were detected under both baseline and stress conditions. The baseline concentration of CRH was higher in FD rats compared with healthy controls (3.88 ± 0.28 vs. 3.55 ± 0.43, *p* < 0.05), which could be downregulated by high-dosage ZZKZ administration (3.54 ± 0.25 vs. 3.88 ± 0.28, *p* < 0.05; Table 5). The FD rats showed a more intense HPA axis response to acute stress with significantly increased levels of CRH, ACTH, and CORT (*p* < 0.01). The stress-induced HPA overactivations in FD rats were significantly suppressed by treating with fluoxetine as well as ZZKZ (Table 5).

## 4. Discussion

The present study provides compelling preclinical evidence that the traditional Chinese medicine formula, Zhizhu Kuanzhong (ZZKZ), could ameliorate anxiety-like and depression-like behaviors in FD rats. Previous clinical evidence has revealed the efficacy of ZZKZ in alleviating FD-related gastrointestinal symptoms such as postprandial fullness, early satiety, belching, and epigastric pain [28,31]. Neonatal irritation with intragastrical iodoacetamide leads to typical FD-like features in rats, such as gastric dysmotility and visceral hypersensitivity [34,35,51], and the reliability of this model in reproducing psychological features, including anxiety and depression, is robustly supported by published papers [35]. In addition to ameliorating gastric hypersensitivity and motor dysfunction, administration of ZZKZ also alleviated anxiety- and depression-like behaviors in FD rats. Our study provides a neurobiological basis for ZZKZ’s clinical benefits and suggests that ZZKZ’s efficacy considerably stems from the modulation of dysfunctional gut–brain interactions. This multi-target effect of ZZKZ on gut–brain interaction is likely attributable to its complex chemical composition, from which we identified four core ingredients: synephrine, naringin, atractylenolide I, and atractylenolide III. Notably, this finding is consistent with the official ingredient specification disclosed by the pharmaceutical manufacturer of the ZZKZ capsule and the *Chinese Pharmacopoeia (2020 Edition)* (https://ydz.chp.org.cn/#/main, accessed on 7 April 2026).

Within the theoretical framework of traditional Chinese medicine, FD is frequently categorized as a syndrome of “*Spleen-deficiency and Qi-stagnation*” based on the principles of holism and syndrome differentiation [52,53]. The principal herbs of the ZZKZ capsule, such as Zhi Shi (*Citrus aurantium* L.), Chai Hu (*Bupleurum chinense* DC.), and Bai Zhu (*Atractylodes macrocephala* Koidz.), are widely recognized as effective prokinetic agents of FGIDs [54,55,56]. Notably, Chai Hu and Bai Zhu have been reported to participate in anti-depression effects and emotional regulation [57,58]. *Atractylodes macrocephala* Koidz. is commonly used for “*liver qi depression* and *liver depression and spleen deficiency*” [59]. *Bupleurum chinense* DC. was a “Sovereign” herb in Chaihu Shugan decoction and Xiaoyao decoction [59,60], both of which were classical prescriptions for relieving depression [57,59,61,62]. This traditional context provides a strong rationale for investigating the neuropsychiatric effects of ZZKZ. The present study bridges this empirical knowledge with modern pharmacology by elucidating the specific molecular mechanisms that underlie these effects. The monoamine theory is widely recognized in the pathogenesis of anxiety and depression [63,64]. Altered levels of monoamine neurotransmitters, such as 5-HT, DA, NE, and gamma-amino butyric acid, in the synaptic cleft of various limbic regions play a crucial role in depressive symptoms [65,66,67]. DA dysfunction in the limbic system has been reported to be associated with the development of anxiety and depression in chronic pain conditions and FD [68]. In alignment with this hypothesis, we identified significant deficiencies of 5-HT, DA, and NE in the hippocampus in FD rats, which were effectively normalized by ZZKZ treatment. Our mechanistic dissection reveals a multi-pronged action on the monoaminergic system in the hippocampus, which determines the levels of monoamines in the synaptic clefts [37]: ZZKZ upregulates the expression of the rate-limiting synthesis enzymes TPH2 and TH while simultaneously downregulating the degradation enzyme MAO_A_ and the serotonin transporter SERT [37,69]. Additionally, ZZKZ administration significantly inhibited the expression of SERT but not DAT and NET in the hippocampus, which potentially inhibits 5-HT reuptake and enhances the duration of serotonergic signaling [22,70]. Among the four core ingredients of ZZKZ (synephrine, naringin, atractylenolide III, and atractylenolide I), synephrine has been identified as a substrate for MAO_A_ in the rat brain [71] and is believed to exhibit β-adrenergic activity [72]. Administration of naringin has also been shown to significantly attenuate doxorubicin-induced elevation of plasma CORT and modulate 5-HT level [73]. Consistently, our molecular docking analysis provides a robust structural basis for ZZKZ’s potential as a direct neuromodulator: the stable hydrogen-bonding networks formed between the core ingredients of ZZKZ and TPH2/TH/MAO_A_/SERT elucidate that ZZKZ facilitates neurotransmitter synthesis while simultaneously inhibiting degradation and synaptic reuptake through regulation of physical interaction and mRNA transcription, thereby restoring the monoaminergic balance in the hippocampus. Besides the monoaminergic system, it is well-established that stress and the consequent dysregulation of the HPA axis are pivotal in the pathogenesis of both FGIDs and psychiatric disorders [74]. The hyperactivation of the HPA axis in response to stress leads to an elevated release of stress hormones, including CRF, ACTH, and CORT [75], as well as β-endorphin (β-EP) [76]. This response is also involved in the occurrence of co-morbid depression and psychological symptoms in patients with FD [35,77]. CRF is a key stress hormone involved in the gut–brain interactions [78], and blocking the CRF1 receptor has shown great therapeutic utility in several preclinical models of depression [79]. Our study demonstrates that FD rats exhibit HPA axis hyper-reactivity, and ZZKZ administration effectively attenuated this maladaptive neuroendocrine stress response. This finding suggests that a second major mechanism of ZZKZ’s therapeutic action involves inhibiting HPA axis hyper-reactivity, thereby enhancing resilience to stress. For the large proportion of FD patients suffering from co-morbid anxiety and depression, ZZKZ offers a promising therapeutic strategy that addresses both the peripheral gastrointestinal distress and the associated psychological burden, potentially reducing the need for high-dose antidepressants, which often carry gastrointestinal side effects [80]. However, several limitations still need to be elucidated. First, our investigation was confined to an FD model where psychiatric symptoms are secondary to gastrointestinal pathology. To confirm the potential antidepressant and anxiolytic properties of ZZKZ, future studies should employ specialized psychiatric animal models (e.g., chronic unpredictable stress) [81]. While our data demonstrate a robust correlation based on neurochemical normalization and behavioral recovery, additional mechanistic research employing pathway-specific interventions is essential to delineate the precise causal underpinnings of ZZKZ’s antidepressant and anxiolytic effects. Second, this study was conducted exclusively in male rats. In this initial exploratory phase, male subjects were selected primarily to minimize the confounding effects of estrous cycle-induced neuroendocrine fluctuations (e.g., estrogen and progesterone) on behavioral performance and HPA axis reactivity [82,83]. However, we recognize this exclusion as a significant limitation. Given the well-documented sex differences in the prevalence of both FD and affective disorders [84], future investigations incorporating female animals are highly warranted to assess potential sex-specific therapeutic effects and mechanisms of ZZKZ. Additionally, the network pharmacology and molecular docking provide primarily predictive insights based on static protein-ligand models and existing databases, which may not fully capture the complex pharmacokinetic profiles or the dynamic “multi-component, multi-target” synergies of ZZKZ in vivo. Therefore, we utilized these computational tools as a preliminary screening mechanism and placed greater emphasis on the subsequent in vivo experimental validation, which consistently shows shifts in neurotransmitter levels and HPA axis markers.

## 5. Conclusions

In conclusion, the present study demonstrates that the ZZKZ capsule effectively ameliorates the disordered gut–brain interactions, mitigating both the gastrointestinal and neuropsychiatric manifestations of functional dyspepsia in a preclinical model. This observed therapeutic efficacy may plausibly be attributed to a dual regulatory mechanism: the normalization of the hippocampal monoaminergic system via concerted regulation of neurotransmitter synthesis, degradation, and transport, and the attenuation of the over-activated hypothalamic–pituitary–adrenal axis response to stress. These findings elucidate the therapeutic efficacy and molecular targets of ZZKZ on co-morbidity depression and anxiety in functional dyspepsia based on gut–brain interaction theory, and they will facilitate the clinical application of ZZKZ as a potential neuromodulator.

## Figures and Tables

**Table 1:** **Composition of the ZZKZ capsule.**

Chinese Name	Scientific Name *	Family	Used Part	Quantity (dry, g) ^#^
Zhi Shi	*Citrus aurantium* L.	Rutaceae	Immature fruit	3.00
Bai Zhu	*Atractylodes macrocephala* Koidz.	Compositae	Rhizome	4.50
Chai Hu	*Bupleurum chinense* DC.	Apiaceae	Root	2.25
Shan Zha	*Crataegus pinnatifida* Bunge	Rosaceae	Mature fruit	2.25

* The Latin names of plants were checked and accepted in the databases of “The Plant List” (http://www.theplantlist.org, accessed on 27 April 2017) and “The World Flora Online” (WFO, http://www.worldfloraonline.org/, accessed on 27 April 2017). ^#^ The dry weight of each raw ingredient per 4.3 g of ZZKZ products.

**Table 2:** **Sequences of PCR reaction primers.**

Gene	Primer Sequences
TPH2	Forward 5′-CCATCGGAGAATTGAAGCAT-3′ Reserve 5′-TTGGAAGGTGGTGATTAGGC-3′
TH	Forward 5′-GTTCATCGGACGGCGACAGA-3′ Reserve 5′-TCCCTACCCTTACGACAAGAGT-3′
SERT	Forward 5′-TCCGCA TGAATGCTGTGTAAC-3′ Reserve 5′-TTGGCTTAGAGGGGAGGAGTC-3′
DAT	Forward 5′-TGGGTTTGGAGTGCTGATTGC-3′ Reserve 5′-GAGGAGACCGAAGCAGCAGAAG-3′
NET	Forward 5′-CATCAACTGTGTTACCAGTTTTATT-3′ Reserve 5′-AAACATGGCCAGAAGAAAGGTACC-3′
MAO_A_	Forward 5′-AGTGGAGTGGCTACATGGAAGGAG-3ʹ Reserve 5′-AGCAGACCAGGCACGGAAGG-3′
MAO_B_	Forward 5′-AGAAGCTCCAGTTGCCTACACG-3′ Reserve 5′-AGAGAAATCTGAGAGTGTTCAT-3′
GAPDH	Forward 5′-ACCACAGTC CATGCCATCAC-3′ Reserve 5′-TCCACCACCCTGTTGCTGTA-3′

TPH2, tryptophan hydroxylase 2; TH, tyrosine hydroxylase; SERT, serotonin transporter; DAT, dopamine transporter; NET, noradrenaline transporter; MAO_A_, monoamine oxidase A; MAO_B_, monoamine oxidase B; GAPDH, glyceraldehyde-3-phosphate dehydrogenase.

**Table 3:** **Body weight and food intake in ZZKZ-treated FD rats.**

	Body Weight (g)			Body Weight Increase (%)	24 h Food Intake (g)
	0 w	7 w	9 w		
HC	16.56 ± 1.07	207.41 ± 7.81	250.01 ± 7.21	20.64 ± 2.38	21.78 ± 2.61
FD	17.53 ± 1.87	188.11 ± 10.55 **	200.29 ± 11.14 ***	6.54 ± 2.77 ***	17.62 ± 2.49 **
FD + FLU	16.73 ± 1.84	187.75 ± 14.11	214.96 ± 13.09 ^#^	14.67 ± 4.52 ^##^	18.44 ± 2.50
FD + ZZKZ-L	15.86 ± 1.05	195.90 ± 15.92	229.05 ± 19.23 ^##^	17.13 ± 7.92 ^##^	19.50 ± 1.58
FD + ZZKZ-M	17.13 ± 1.42	185.49 ± 9.98	220.53 ± 14.54 ^##^	18.94 ± 5.85 ^###^	22.47 ± 1.67 ^###^
FD + ZZKZ-H	16.19 ± 1.89	178.57 ± 10.14	209.46 ± 12.05	15.44 ± 8.46 ^#^	21.09 ± 2.01 ^#^

Data are shown as mean ± SD, *n* = 6–8. ** *p* < 0.01, and *** *p* < 0.001 FD group vs. HC group; ^#^ *p* < 0.05, ^##^ *p* < 0.01, and ^###^ *p* < 0.001 FD + FLU/FD + ZZKZ group vs. FD group.

**Table 4:** **Docking results of ZZKZ’s core ingredients with monoaminergic molecules.**

Compound Name	Affinity (kcal/mol)			
	TPH2	TH	MAO_A_	SERT
synephrine	−6.1	−5.7	−6.9	−6.0
naringin	−9.9	−9.2	−9.2	−10.1
atractylenolide III	−8.6	−6.7	−8.0	−9.5
atractylenolide I	−8.8	−7.1	−7.8	−7.4

**Table 5:** **The HPA axis response to stress in FD rats.**

Group	CRH	ACTH	CORT
Baseline HPA axis activity (pg/mL)			
HC	3.55 ± 0.43	22.50 ± 3.37	170.48 ± 25.03
FD	3.88 ± 0.28 *	23.96 ± 2.94	172.16 ± 24.32
FD + FLU	3.68 ± 0.45	22.26 ± 3.25	165.56 ± 24.21
FD + ZZKZ L	3.90 ± 0.28	24.03 ± 1.81	178.69 ± 13.46
FD + ZZKZ M	3.62 ± 0.37	23.49 ± 3.54	174.67 ± 26.24
FD + ZZKZ H	3.54 ± 0.25 **^#^**	22.79 ± 2.18	169.49 ± 16.20
HPA axis response to stress (pg/mL)			
HC	4.68 ± 0.57	41.20 ± 6.19	263.08 ± 38.52
FD	6.11 ± 0.46 ***	55.84 ± 6.84 ***	339.15 ± 47.88 **
FD + FLU	4.86 ± 0.59 **^##^**	40.85 ± 6.48 **^###^**	296.79 ± 22.37 **^#^**
FD + ZZKZ L	6.19 ± 0.48	53.86 ± 4.07	343.35 ± 50.20
FD + ZZKZ M	5.26 ± 0.54 **^#^**	44.59 ± 6.68 **^##^**	314.50 ± 32.35
FD + ZZKZ H	4.66 ± 0.34 **^###^**	47.85 ± 4.55 **^#^**	288.12 ± 37.73 **^#^**

Data were shown as mean ± SD, *n* = 6–8. * *p* < 0.05, ** *p* < 0.01, and *** *p* < 0.001 FD group vs. HC group; ^#^ *p* < 0.05, ^##^ *p* < 0.01, and ^###^ *p* < 0.001 FD + FLU/FD + ZZKZ group vs. FD group.

## Data Availability

The data presented in this study are available on request from the corresponding author. The data are not publicly available due to the fact that the investigational drug in this study contains commercial drug information, and the authors do not wish the research results to be misinterpreted or misused.

## Abbreviations

5-HIAA	5-hydroxyindoleacetic acid
5-HT	5-Hydroxytryptamine
ACTH	adrenocorticotropic hormone
CORT	corticosterone
CRH	corticotropin-releasing hormone
DA	dopamine
DAT	dopamine transporter
DOPAC	3,4-dihydroxyphenylacetic acid
ELISA	enzyme-linked immunosorbent assay
FD	functional dyspepsia
FGIDs	functional gastrointestinal disorders
FLU	fluoxetine
FST	forced swimming test
GO	Gene Ontology
HC	healthy control
HPA	hypothalamic–pituitary–adrenal axis
HPLC	high-performance liquid chromatography
HVA	homovanillic acid
KEGG	Kyoto Encyclopedia of Genes and Genomes
LDB	light dark box test
MAO_A_	monoamine oxidase A
MAO_B_	monoamine oxidase B
NE	norepinephrine
NET	noradrenaline transporter
OFT	open field test
SERT	serotonin transporter
SPT	sucrose preference test
TH	tyrosine hydroxylase
TPH2	tryptophan hydroxylase 2
ZZKZ	Zhizhu Kuanzhong
